# Hypoxia Inducible Factor 1α Promotes Endogenous Adaptive Response in Rat Model of Chronic Cerebral Hypoperfusion

**DOI:** 10.3390/ijms18010003

**Published:** 2017-01-17

**Authors:** Ying Yang, Jieyang Ju, Min Deng, Jing Wang, Hui Liu, Li Xiong, Junjian Zhang

**Affiliations:** 1 Department of Neurology, Zhongnan Hospital, Wuhan University, Donghu Road 169#, Wuhan 430071, China; 200432180213@whu.edu.cn (Y.Y.); julia88317@sohu.com (J.J.); minmin0jiejie@126.com (M.D.); wangjing871207@126.com (J.W.); liuhui0070@gmail.com (H.L.); may.lixiong@gmail.com (L.X.); 2 Department of Neurology, Zhejiang Provincial People’s Hospital, Shangtang Road 158#, Hangzhou 310014, China

**Keywords:** chronic cerebral hypoperfusion, hypoxia inducible factor 1α (*HIF-1α*), neuroprotective, cognition

## Abstract

Hypoxia inducible factor 1α (*HIF-1α*), a pivotal regulator of gene expression in response to hypoxia and ischemia, is now considered to regulate both pro-survival and pro-death responses depending on the duration and severity of the stress. We previously showed that chronic global cerebral hypoperfusion (CCH) triggered long-lasting accumulation of HIF-1α protein in the hippocampus of rats. However, the role of the stabilized HIF-1α in CCH is obscure. Here, we knock down endogenous *HIF-1α* to determine whether and how HIF-1α affects the disease processes and phenotypes of CCH. Lentivirus expressing HIF-1α small hairpin RNA was injected into the bilateral hippocampus and bilateral ventricles to knock down *HIF-1α* gene expression in the hippocampus and other brain areas. Permanent bilateral common carotid artery occlusions, known as 2-vessel occlusions (2VOs), were used to induce CCH in rats. Angiogenesis, oxidative stress, histopathological changes of the brain, and cognitive function were tested. Knockdown of *HIF-1α* prior to 2VO significantly exacerbates the impairment of learning and memory after four weeks of CCH. Mechanically, reduced cerebral angiogenesis, increased oxidative damage, and increased density of astrocytes and microglia in the cortex and some subregions of hippocampus are also shown after four weeks of CCH. Furthermore, *HIF-1α* knockdown also disrupts upregulation of regulated downstream genes. Our findings suggest that *HIF-1α*-protects the brain from oxidative stress and inflammation response in the disease process of CCH. Accumulated HIF-1α during CCH mediates endogenous adaptive processes to defend against more severe hypoperfusion injury of the brain, which may provide a therapeutic benefit.

## 1. Introduction

Chronic crebral hypoperfusion (CCH) is a prevalent pathophysiological state in patients with Alzheimer’s disease (AD) and vascular dementia (VaD). CCH has been identified as one of the initial conditions that are critical to the development of cognitive dysfunction [1]. Current studies have indicated that deranged energy metabolism, glial activation, apoptosis, oxidative stress, neuronal damage, and white matter lesions caused by cerebral hypoperfusion might be the pathophysiological mechanisms that contribute to cognitive impairment [2,3]. Despite the detrimental effects of hypoperfusion, the decline in cellular oxygen levels and energy supplies also induces compensatory or endogenous adaptive mechanisms to defend against hypoperfusion injury and to allow recovery of brain function [2,4]. In agreement, gradual recovered cerebral blood flow (CBF), increased capillary diameter, neovascularization, and enhanced expression of vascular endothelial growth factor (*VEGF*) were noted in the cortex and hippocampus in rodent models of CCH [2], which indicates the compensatory or adaptive mechanisms. Because of the multifactorial pathogenesis of dementia, a large number of clinical trials which sought to identify protective strategies against this disease have failed, so the recruitment of endogenous neuroprotective pathways represents a potential strategy for developing therapeutics for the cognitive impairment. The regulation of the endogenous adaptive and neuroprotective processes involves the concerted activation of various transcription factors. In the condition of CCH, hypoxia inducible factor 1 (*HIF-1*) is one of the most important transcription factors involved in endogenous adaptive response.

*HIF-1* is a pivotal regulator of gene expression in response to hypoxia or ischemia. It is composed of an oxygen-regulated subunit known as *HIF-1α* and a constitutively expressed *HIF-1β* subunit. In most cases, *HIF-1* activity is controlled by the availability and activity of the *HIF-1α* subunit. Under normoxic conditions, *HIF-1α* is hydroxylated and rapidly degraded. Under hypoxic-ischemic conditions, hydroxylases are less active, thus the *HIF-1* complex accumulates and translocates to the nucleus, which results in the transcription of *HIF-1* target genes. *HIF-1* target genes include pro-survival genes involved in angiogenesis, energy metabolism, erythropoiesis, vasomotor control, and cell proliferation, such as vascular endothelial growth factor (*VEGF*), glucose transporter-1 (*GLUT-1*), and erythropoietin (*EPO*), which, in turn, promotes neuronal adaptation for survival under hypoxic conditions [5,6].

Neuron-specific knockdown of *HIF-1α* aggravates brain damage after a 30 min middle cerebral artery occlusion (MCAO) and reduces the survival rate of MCAO mice [7]. Inhibiting *HIF-1α* hydroxylases to trigger the accumulation of HIF-1α protects the brain from cerebral stroke damage [8,9]. Thus, *HIF-1α* appears to be a potential target for the development of novel therapeutic interventions in ischemia-related neurological disease.

Paradoxically, *HIF-1α* is also involved in hypoxia-dependent inflammation, apoptosis, and cellular stress. *HIF-1α* governs the expression of several pro-apoptotic genes including Bcl-2/adenovirus EIB 19 kDa-interacting protein 3 (*BNIP-3*), NADPH oxidase activator 1 (*Noxa*), NIP3-like protein X (*NIX*) [10,11,12]. Brain-specific knockout of *HIF-1α* may be neuroprotective after acute global ischemic injury for 75 min, and genes involved in apoptotic pathways are downregulated in *HIF-1* knockout mice [13].

Consequently, both neuroprotective and detrimental effects of *HIF-1α* have been observed in animal models of acute ischemia [14]. The role for *HIF-1α* in mediating either pro-death or pro-survival responses may depend on the severity and duration of the ischemia insult [15] and the types of pathological stimuli [16]. Mild hypoxia induced adaptive gene expression can occur transiently or persistently depending on the duration of the stimulus, whereas with severe or sustained hypoxic conditions *HIF-1α* can promote apoptotic cell death [17].

CCH is a less severe form of ischemia that develops more slowly than acute ischemic stroke [18,19], therefore, *HIF-1α* may play a more important role in this pathological process. Our previous study has shown that chronic global cerebral hypoperfusion triggered long-lasting accumulation of *HIF-1α* protein in rat hippocampus [20]. However, the role of the stabilized *HIF-1α* during CCH is still obscure. The aim of this study was to investigate the role of endogenous *HIF-1α* stabilization during CCH in rats.

## 2. Results

### 2.1. Lentiviral-Mediated shRNAi Delivery Leads to Efficient Transfection and Knockdown of HIF-1α in Some Brain Areas of Rats

To ascertain the role of endogenous *HIF-1α* accumulation during CCH in rats, we used a loss of function approach by silencing *HIF-1α* expression using lentivirus-mediated shRNAi (short-hairpin RNA interference) against *HIF-1α* mRNA (pLVx-GFP-shHIF-1α). The green fluorescence protein (GFP) was used to examine lentiviral transfection efficiency by microscopy.

**Figure 1:**
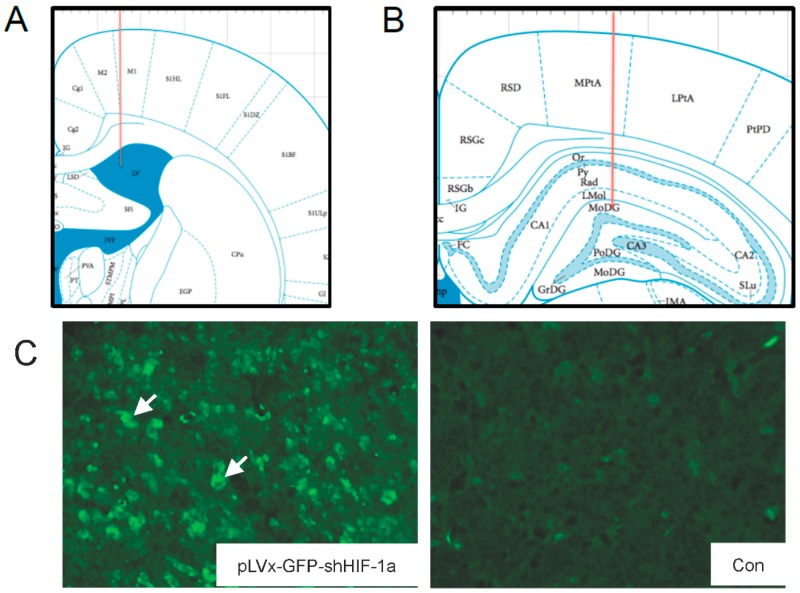

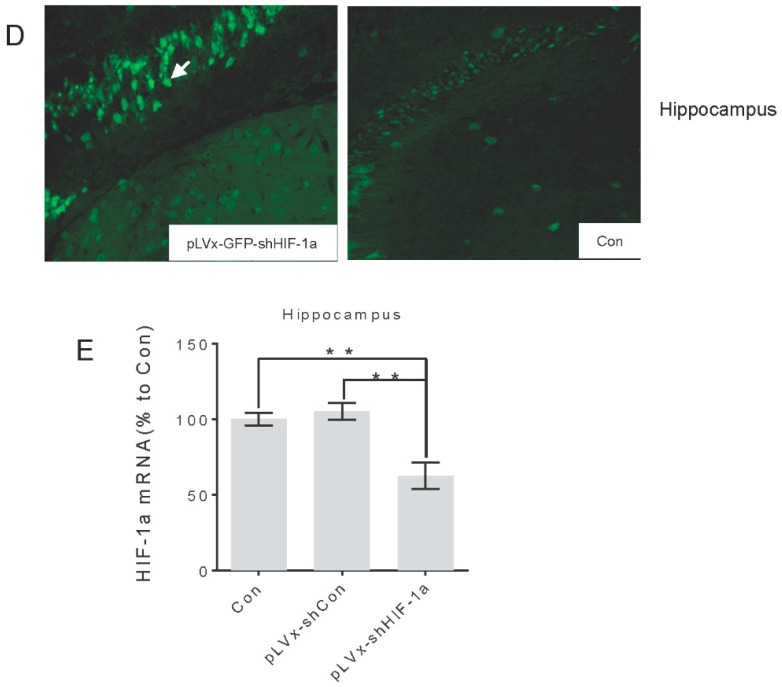
The delivery efficiency of lentivirus vectors and its efficacy in reducing hypoxia inducible factor 1α (*HIF-1α*) mRNA expression in normal rats one week after stereotaxic injection. (**A**,**B**) Injection sites in ventricle and hippocampus. Red lines represent needle tracks; (**C**) GFP+ cells (arrows) in cortex of pLVx-GFP-shHIF-1α-injected rat (**left**) and saline-injected control rat (**right**); (**D**) GFP+ cells (arrow) in hippocampus (DG subregion) of pLVx-GFP-shHIF-1α-injected rat (**left**) and saline-injected control rat (**right**); (**E**) qRT-PCR analysis of *HIF-1α* mRNA levels in hippocampus one week after stereotaxic injection (Con: saline-injected rats; pLVx-shCon: pLVx-GFP-shCon-injected rats; pLVx-shHIF-1α: pLVx-GFP-shHIF-1α-injected rats). Values for *HIF-1α* mRNA were normalized to β-actin. Values are expressed as a percentage of the average value for saline-injected rats (Con) and are means ± SEM (*n* = 4). ** *p* < 0.01 (one-way ANOVA followed by Bonferroni post hoc tests).

One week after the stereotaxic injection into the bilateral hippocampus and bilateral ventricles, lentiviral vectors could effectively transfect most parts of the hippocampus and cortex (Figure 1C,D). Dense GFP positive cells were also detected in brain regions surrounding ventricles (Not shown in our figure), while few GFP positive cells could be seen in the corpus callosum (Not shown in our figure). This efficient transfection in the hippocampus and cortex correlated with efficacy in reducing HIF-1α mRNA expression. The result showed that *HIF-1α* mRNA expression decreased by around 40% in the hippocampus of normal rats one week post-transfection by pLVx-GFP-shHIF-1α (*p* < 0.01; Figure 1E). It is confirmed that HIF-1α expression was downregulated before hypoperfusion, since 2-vessel occlusions (2VO) surgery was performed one week after the transfection in our study.

**Figure 2:**
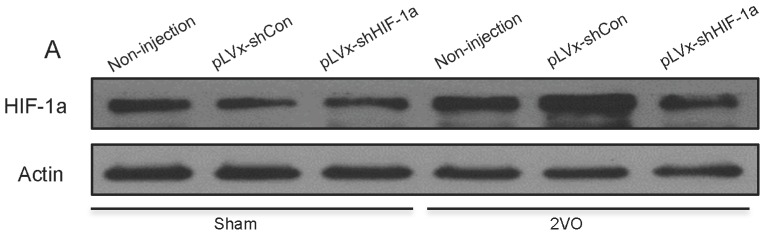

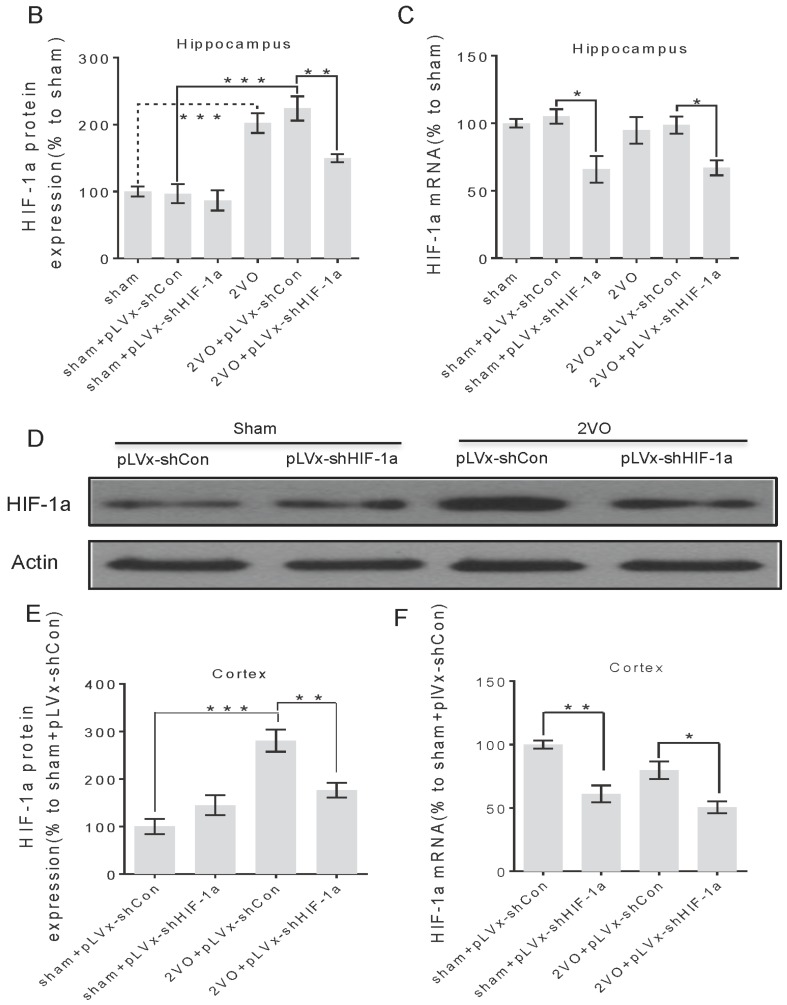
The effects of lentiviral-mediated shRNAi on *HIF-1α* mRNA expression and protein accumulation under four-week hypoperfusion. (**A**,**B**) Western blot analysis of *HIF-1α* protein levels in hippocampus of 2-vessel occlusions (2VO) or sham rats that received stereotaxic injection of pLVx-GFP-shCon or pLVx-GFP-shHIF-1α. As blank control, 2VO or sham rats without stereotaxic injection were also included in our study; (**C**) qRT-PCR analysis of *HIF-1α* mRNA levels in hippocampus of rats in each group; (**D**,**E**) Western blot analysis of *HIF-1α* protein levels in cortex of rats in each group; (**F**) qRT-PCR analysis of *HIF-1α* mRNA levels in cortex of rats in each group. Values for *HIF-1α* protein and mRNA were normalized to β-actin protein or mRNA, respectively. Values are expressed as a percentage of the average value for the first group on the left and are means ± SEM (*n* = 4–5). * *p* < 0.05 and ** *p* < 0.01, *** *p* < 0.001 (one-way ANOVA followed by Bonferroni post hoc tests).

Four weeks after 2VO or sham surgery, we measured *HIF-1α* mRNA and protein expressions in the hippocampus and cortex. *HIF-1α* mRNA could still be reduced by pLVx-GFP-shHIF-1α in 2VO and sham rats (Figure 2C,F), which indicated that our vectors could transfect neurons stably for at least five weeks. In addition, results showed that four weeks after 2VO, the accumulation of *HIF-1α* protein was significantly increased compared with sham rats, while pLVx-GFP-shHIF-1α reduced the endogenous *HIF-1α* protein accumulations in 2VO by approximately 40% (Figure 2A,B,D,E). The control pLVx-GFP-shCon vector did not modify *HIF-1α* expressions. Thus, lentiviral-mediated shRNAi reduced *HIF-1α* protein levels under hypoperfusion.

### 2.2. Knockdown of HIF-1α Aggravated Spatial Learning and Memory Deficits Induced by CCH

Once we determined that pLVx-GFP-shHIF-1α prevents *HIF-1α* protein accumulation in the hippocampus and cortex under hypoperfusion, we explored the effects of reducing *HIF-1α* accumulation on CCH-induced cognitive impairment.

The rats were subjected to five consecutive days of training in the Morris Water Maze (MWM) to investigate their spatial learning abilities four weeks after 2VO or sham surgery with or without HIF-1α-knockdown treatment. By analyzing the escape latency to the platform day by day, results indicated that beginning on day 3, the 2VO + pLVx-GFP-shCon rats took a longer time to find the platform compared with sham + pLVx-GFP-shCon rats (Figure 3B day 3, *p* < 0.05; day 4, *p* < 0.01). Consistently, the 2VO rats (without stereotaxic injection) required a significantly longer time to find the hidden platform than the sham rats (Figure 4A day 4, *p* < 0.05; day 5, *p* < 0.05). These findings are consistent with those from previous studies that show that CCH induced by 2VO could cause spatial learning ability impairment. Furthermore, the hypoperfused rats with pLVx-GFP-shHIF-1α-transfection (2VO + pLVx-GFP-shHIF-1α) took even more time to find the platform than 2VO + pLVx-GFP-shCon rats (Figure 3B day 4–5, *p* < 0.05).

**Figure 3:**
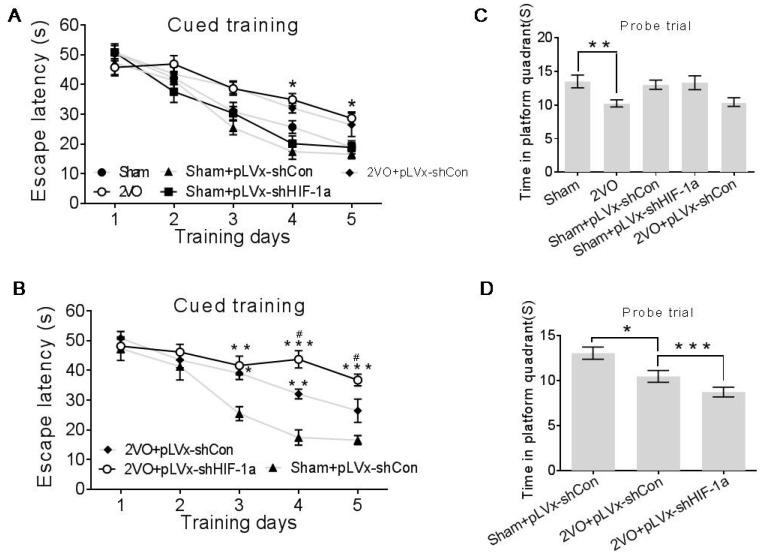
The effects of *HIF-1α* knockdown on CCH-induced deficits in spatial learning and memory as measured with the Morris Water Maze. (**A**,**B**) Mean daily escape latencies (i.e., times from the start to the hidden platform) during cued training; * *p* < 0.05 compared to 2VO in (**A**); * *p* < 0.05, ** *p* < 0.01, *** *p* < 0.001 compared to sham+pLVx-GFP-shCon, ^#^ *p* < 0.05 compared to 2VO + pLVx-GFP-shCon in (**B**) (Two-way ANOVA followed by Bonferroni post-tests); (**C**,**D**) times spent in the target quadrant during the probe trials. * *p* < 0.05, ** *p* < 0.01, *** *p* < 0.001. (One-way ANOVA followed by Bonferroni post hoc tests, or unpaired student's *t* test). All values are expressed as mean ± SEM (*n* = 8–16).

**Figure 4:**
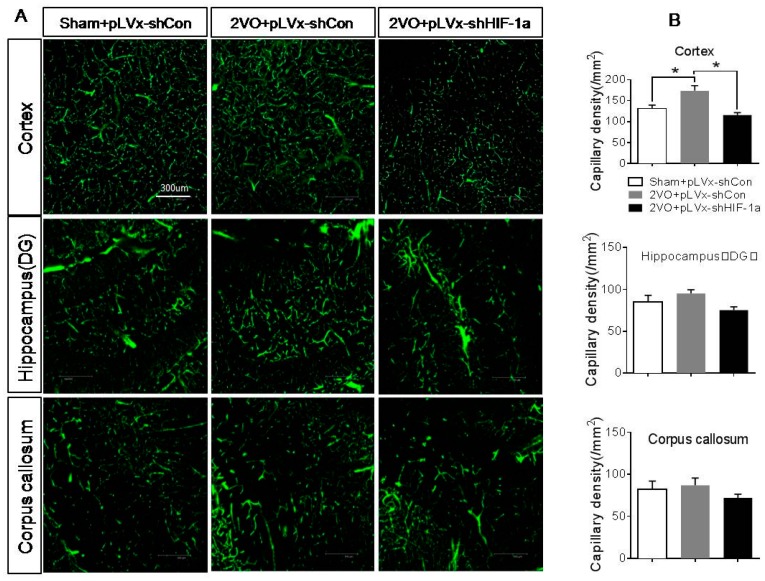
The effects of *HIF-1α* knockdown on CCH-induced angiogenesis. (**A**) Representative images of FITC-dextran-perfused vessels in the hippocampus, cortex, and corpus callosum four weeks after 2VO or sham surgery with or without HIF-1α-knockdown treatment. Bar = 300 µm (the magnification of all images is consistent); (**B**) the quantitative capillary density. Capillary density was expressed as the number of capillaries (diameter <12 µm) per square millimeter. Values are expressed as mean ± SEM (*n* = 4). * *p* < 0.05 (One-way ANOVA followed by Bonferroni post hoc tests).

In the probe trials after the platform was removed, spatial memory was evaluated by measuring the time spent in the target quadrant. As shown in Figure 4C,D, the 2VO + pLVx-GFP-shCon (or 2VO) rats spent less time in the platform region than did the sham + pLVx-GFP-shCon rats (or sham rats) (Figure 4D, *p* < 0.05; Figure 4C, *p* < 0.01). The time spent in the platform region was also shorter for the 2VO + pLVx-GFP-shHIF-1α rats than sham + pLVx-GFP-shCon rats (Figure 3D, *p* < 0.001), and the gap was much greater. These results indicate that reducing *HIF-1α* accumulation during CCH aggravated the spatial learning and memory deficits induced by CCH.

The control pLVx-GFP-shCon vector did not affect MWM results in either 2VO or sham rats (Figure 3A,C).

### 2.3. Knockdown of HIF-1α Reduced CCH-Induced Angiogenesis

Increased neovascularization in the cortex and hippocampus during CCH is one of the compensatory or adaptive mechanisms of the brain. Genes regulated by *HIF-1α* (*VEGF* and *EPO*) play a critical role in angiogenesis, therefore, exploring the effects of *HIF-1α* knockdown on CCH-induced angiogenesis could help to figure out how *HIF-1α* affects cognitive function in CCH.

Fluorescein-isothiocyanate (FITC)-dextran-perfused vessels in the hippocampus, cortex, and corpus callosum are shown in Figure 4. Four weeks after 2VO, there was a significant increase in the density of capillaries in the cortex of 2VO + pLVx-GFP-shCon rats compared with sham + pLVx-GFP-shCon rats (*p* < 0.05). Such an increase in capillaries was suppressed by pLVx-GFP-shHIF-1α, as the results showed that the density of capillaries in 2VO + pLVx-GFP-shHIF-1α rats was much less than in 2VO + pLVx-GFP-shCon rats (*p* < 0.05). No significant change was observed in the corpus callosum and hippocampus.

### 2.4. Knockdown of HIF-1α Increased CCH-Induced Oxidative Damage

Oxidative stress has been regarded as an essential factor in the development of CCH-related cognitive impairment. The *4-HNE* (4-hydroxynonenal)-modified protein can be used as a marker of oxidative neuronal damage [21].

**Figure 5:**
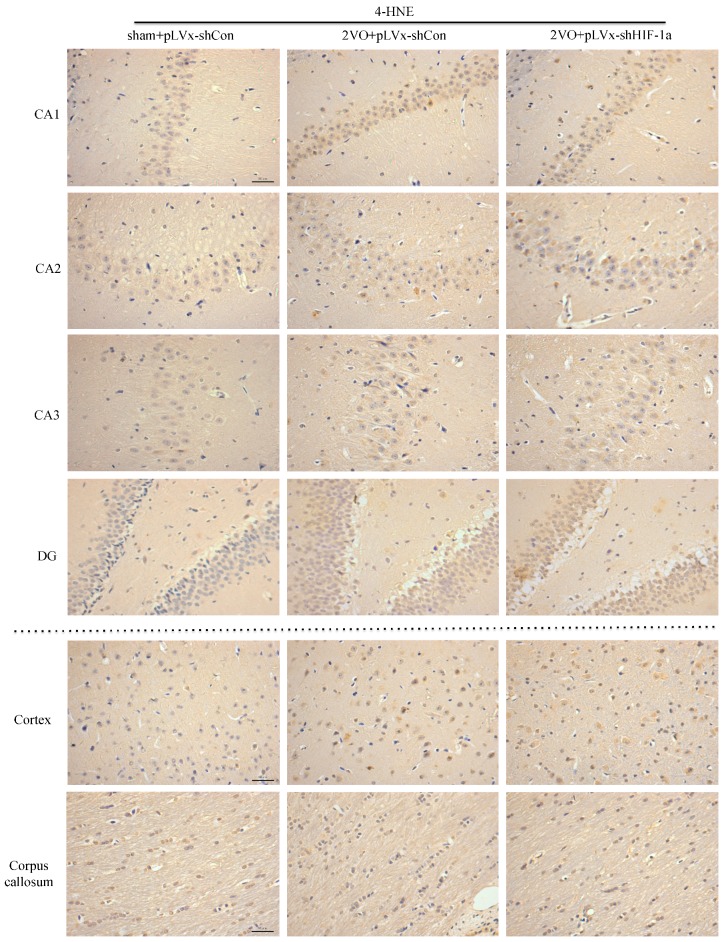

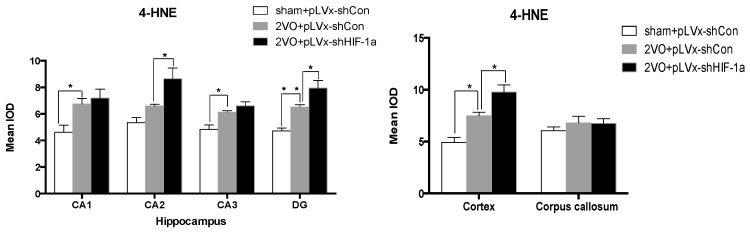
The effects of *HIF-1α* knockdown on CCH-induced lipid peroxidation. Representative photomicrographs of *4-HNE* immunohistochemistry in cerebral cortex, corpus callosum and four subregions of the hippocampus four weeks after sham or 2VO surgery with or without *HIF-1α*-knockdown treatment. The positive cells are dyed brown. Bar = 50 µm (the magnification of all images is consistent). The histograms represent the quantitative results of *4-HNE* immunohistochemistry. The bars represent the mean IOD (integral optical density) ± SEM (*n* = 6). * *p* < 0.05, ** *p* < 0.01. (One-way ANOVA followed by Bonferroni post hoc tests).

The results of immunohistochemical analyses of *4-HNE* in the hippocampus (CA1, CA2, CA3, and DG subregions), cortex, and corpus callosum are shown in Figure 5. Four weeks after 2VO or sham surgery, there were significant increases in *4-HNE* levels in the cortex and some (CA1, CA3, DG) subregions of the hippocampus in pLVx-GFP-shCon-transfected 2VO compared with pLVx-GFP-shCon-transfected sham rats (*p* < 0.05 or *p* < 0.01). Further increases in *4-HNE* levels in CA2 and DG subregions of the hippocampus were found in pLVx-GFP-shHIF-1α-transfected 2VO rats compared with pLVx-GFP-shCon-transfected 2VO rats (*p* < 0.05). No significant change was observed in the corpus callosum following 2VO and HIF-1α-knockdown treatment.

### 2.5. Knockdown of HIF-1α Increased Glial Activation Induced by CCH

**Figure 6:**
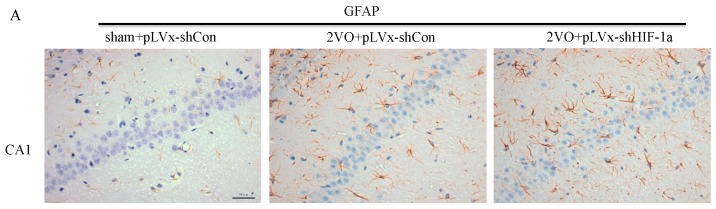

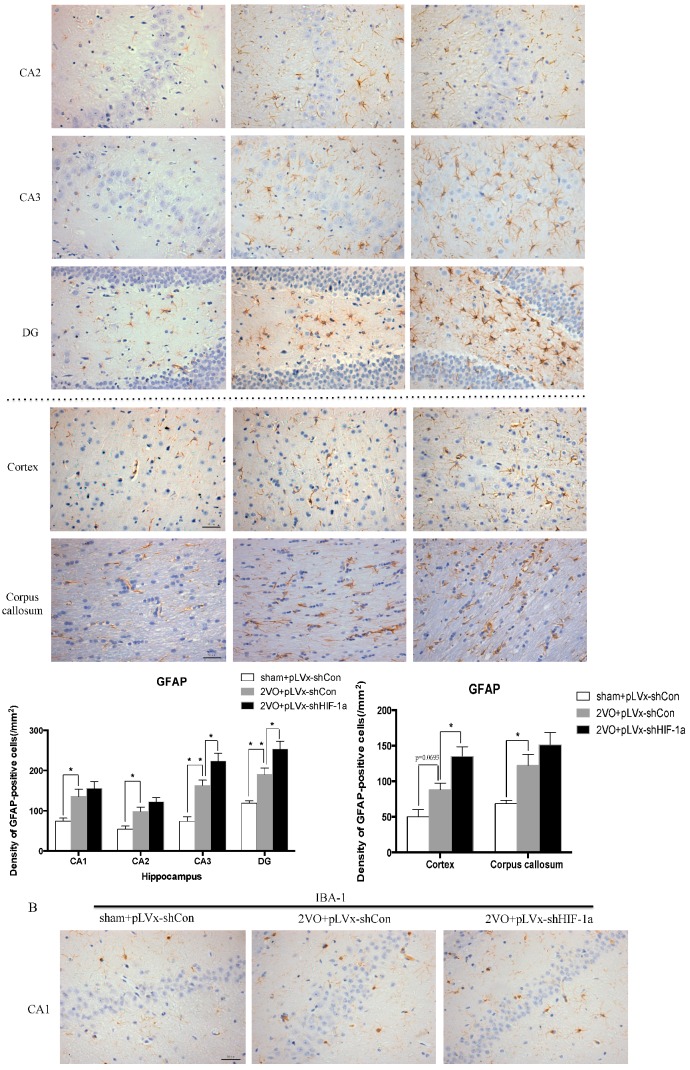

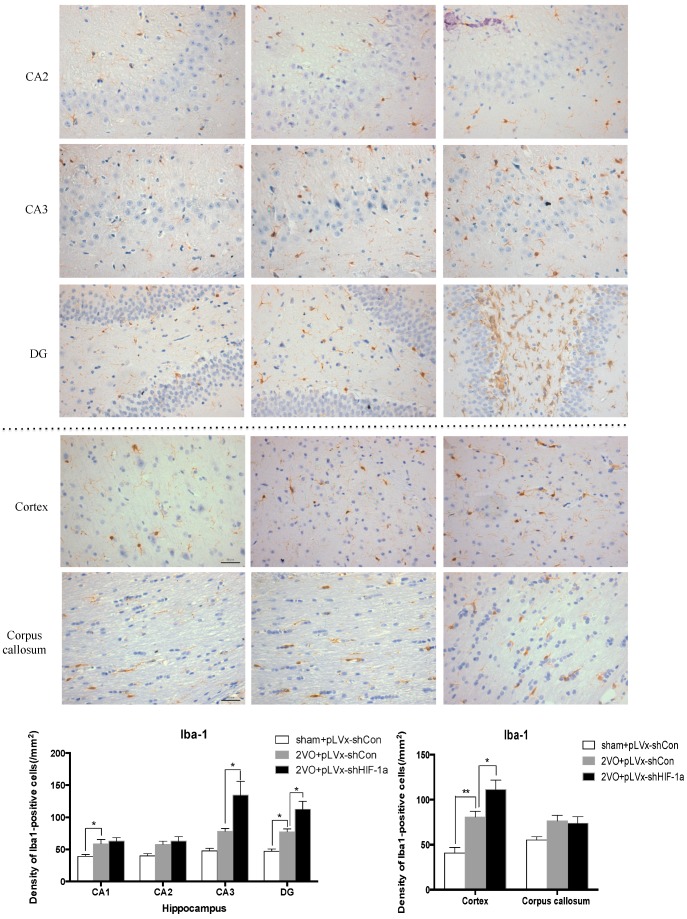
The effects of *HIF-1α* knockdown on CCH-induced glial activation. Representative photomicrographs of glial fibrillary acidic protein (*GFAP*) (**A**) and Iba-1 (**B**) immunohistochemistry in cortex, corpus callosum, and four subregions of the hippocampus four weeks after sham or 2VO surgery with or without *HIF-1α*-knockdown treatment. The positive cells are dyed brown. Bar = 50 µm (the magnification of all images is consistent). The Histograms showing the densities of the cells that were immunoreactive for *GFAP* (**A**) and *Iba-1* (**B**) (per mm^2^). The bars represent the mean ± SEM (*n* = 8). * *p* < 0.05, ** *p* < 0.01 (One-way ANOVA followed by Bonferroni post hoc tests).

Increasing evidence indicates that inflammatory-related microglia and astrocytes are involved in cognitive impairment during CCH. Immunohistochemistry studies were performed to evaluate microglia and astrocyte responses after 2VO and *HIF-1α*-knockdown treatment (Figure 6).

Results revealed that four weeks after 2VO or sham surgery, the densities of glial fibrillary acidic protein (GFAP)-positive astrocytes in the hippocampus (*p* < 0.05 or *p* < 0.01; Figure 6A,B), cortex (*p* = 0.0693), and corpus callosum (*p* < 0.05) were significantly greater in pLVx-GFP-shCon-transfected 2VO rats than in pLVx-GFP-shCon-transfected sham rats. Moreover, additional increases of GFAP-positive astrocytes in the hippocampus (CA3 and DG subregions) and cortex were found in pLVx-GFP-shHIF-1α-transfected 2VO rats compared with pLVx-GFP-shCon-transfected 2VO rats (*p* < 0.05, Figure 6A). These results indicate that HIF-1α knockdown promoted a 2VO-induced increase in astrocyte activation.

Similarly, 2VO caused a large increase in *Iba-1*-immunopositive microglial activation in the hippocampus (CA1, CA3, and DG subregions) and cortex (*p* < 0.05, *p* < 0.01; Figure 6B). With *HIF-1α* knockdown, 2VO-induced increases in the number of Iba-1-positive microglia were even higher in the cortex and hippocampus (CA3 and DG subregions).

As a result, the lack of *HIF-1α* increases CCH-induced inflammatory-related glial activation.

### 2.6. The Effects of HIF-1α Knockdown on the Expression of Downstream Genes During CCH

As a transcription factor, *HIF-1* plays a role in regulating downstream genes during hypoxia or ischemia. We measured the expressions of *HIF-1* target genes (pro-survival and pro-apoptosis genes) in the hippocampus four weeks following the 2VO or sham operation with or without *HIF-1α* knockdown.

**Figure 7:**
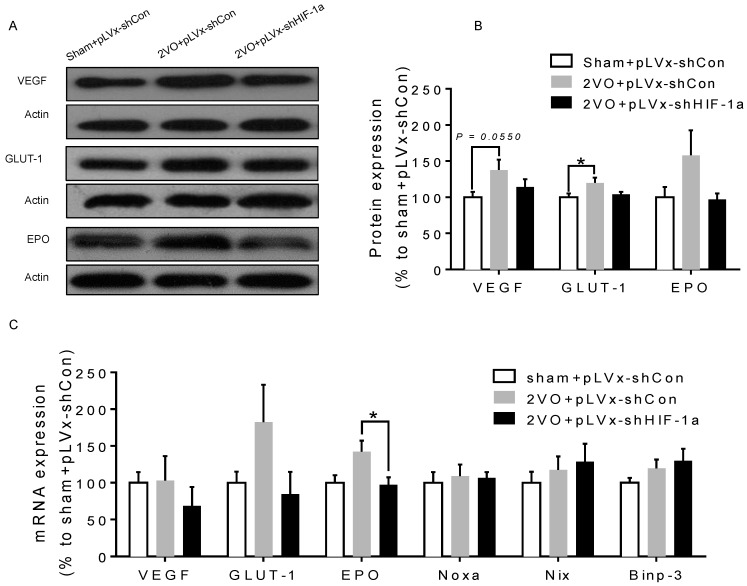
The effects of *HIF-1α* knockdown on the expression of downstream genes during CCH. (**A**,**B**) Western blot analysis of *VEGF/GLUT-1/EPO* protein levels in hippocampus four weeks following 2VO or sham operation with or without HIF-1α knockdown; (**C**) qRT-PCR analysis of *VEGF/GLUT-1/EPO* and *Noxa/Nix/Binp*-3mRNA levels in hippocampus of rats in each group. Values for protein and mRNA were normalized to β-actin protein or mRNA, respectively. Values are expressed as a percentage of the average value for sham + pLVx-GFP-shCon rats and are mean ± SEM (*n* = 4–5). * *p* < 0.05 (One-way ANOVA followed by Bonferroni post hoc tests).

Western blot analysis showed that protein levels of pro-survival genes (*VEGF, GLUT-1*) significantly increased four weeks after 2VO compared with sham rats (*p* = 0.0550, *p* < 0.05; Figure 7A,B). Such increases were attenuated in pLVx-GFP-shHIF-1α-transfected 2VO rats (Figure 7A,B).

qRT-PCR analysis indicated that the mRNA levels of pro-survival genes showed a statistically insignificant increasing trend four weeks after 2VO (*p* > 0.05; Figure 7C), which is in accordance with our previous report that *HIF-1* downstream genes were only significantly upregulated during the first week after 2VO [20]. However, the mRNA levels of pro-survival genes (especially *EPO*) were reduced in pLVx-GFP-shHIF-1α-transfected 2VO rats compared with pLVx-GFP-shCon-transfected 2VO rats (*p* < 0.05; Figure 7C). What is notable is that no significant changes were observed in mRNA levels of pro-apoptosis genes (*Noxa*, *Nix*, *Binp-3*) after 2VO and *HIF-1α* knockdown (Figure 7C).

## 3. Discussion

We previously reported that CCH induced by 2VO triggered a robust and long-lasting *HIF-1α* protein accumulation in rats: both pro-apoptotic and pro-survival downstream genes were upregulated only during the early stage after 2VO. Based on this finding, we further investigated the role of *HIF-1α* in the chronic stage following 2VO. To this end, we knocked down *HIF-1α* using lentivirus-mediated shRNAi against *HIF-1α* mRNA. The results demonstrated that the downregulation of *HIF-1α* accumulation during 2VO reduced the compensatory angiogenesis, and aggravated neuronal damage and cognitive impairment induced by 2VO.

### 3.1. Lentivirus-Mediated shRNAi Lowers the Expression of HIF-1α In Vivo and Reduces the Accumulation of HIF-1α during CCH

RNA interference (RNAi) has been established as a powerful tool to characterize gene function in mammals [22]. RNAi is triggered by the presence of double-stranded RNA in the cell and results in rapid destruction of the mRNA containing the identical sequence, exerting its gene-silencing activity. Recombinant lentiviral vector is particularly useful for gene therapy in CNS injury because of its safety and stability. In this study, we knocked down *HIF-1α* using shRNAi (short-hairpin RNAi) against *HIF-1α* mRNA, introduced into brain cells using lentivirus. The lentivirus could efficiently transfect bilateral cortices and hippocampus one week after being injected into the bilateral hippocampus and ventricles, and lower *HIF-1α* mRNA expression one week post-transfection. It ensures that the *HIF-1α* mRNA expression can be decreased before treating rats with 2VO operation. Moreover, lentiviral-mediated shRNAi can reduce endogenous *HIF-1α* protein accumulation under hypoperfusion. 

### 3.2. HIF-1α Accumulation during CCH Protects against Cognitive Impairment via Its Protective Effects

Our results suggest that reducing *HIF-1α* accumulation during CCH suppressed CCH-induced angiogenesis in the cortex, and enhanced oxidative damage and glial activation in the cortex and hippocampus. Moreover, it aggravated the CCH-induced spatial learning and memory deficits.

Considerable evidence indicates that CCH contributes to the development and progression of dementia through pathways of inflammation and oxidative stress and results in damage to learning and memory functions [23]. Oxidative stress has been regarded as a common pathophysiological change and an essential factor in the development of CCH-related cognitive impairment [3]. Studies focused on the neuroprotective effects of antioxidants indicated that antioxidants could attenuate oxidative damage in rats’ brains and reverse the cognitive deficits induced by CCH [24,25,26]. Inflammatory-related microglia and astrocytes are also considered to be involved in the cognitive impairment in CCH [27]. Previous studies have demonstrated that chronic cerebral hypoperfusion could induce the activation of inflammatory glial cells [28]. These inflammatory glial cells are directly toxic to neurons, which causes particularly severe damage to the central nervous system [29]. Consistent with previous reports, our study verified that CCH by 2VO triggered oxidative damage and glial activation. Moreover, when HIF-1α protein accumulation in CCH was reduced, the oxidative damage and glial activation were even more severe. HIF-1α regulated downstream genes *VEGF* and *EPO* have direct neurotrophic effects and can prevent neuronal injury due to oxidative stress evoked by hypoxia or ischemia and can enhance recovery following stroke [16]. *GLUT-1* is also a *HIF-1α* target gene which could enhance the concentration of glucose and thereby ameliorate neuroprotection. Glucose transport and glycolytic flow as a result of *HIF-1α* activation by hypoxia has been linked to cell survival [30]. Thus, we concluded that *HIF-1α* is required for neuroprotection from CCH.

Besides the detrimental effects caused by CCH, some compensatory and endogenous responses can also be induced during CCH in order to adapt to the decreased perfusion. As our study indicated, the density of perfused cerebral capillaries was increased after 2VO, especially in the cortex. During cerebral ischemia, the damaged tissue tries to increase oxygen delivery by the induction of angiogenesis. *HIF-1α*-regulated cytokines (*VEGF* and *EPO*), which have been implicated in angiogenesis, were increased after CCH as well. The angiogenesis induced by CCH was decreased in *HIF-1α* knockdown rats which further confirmed that *HIF-1α* is involved in endogenous adaptive response.

Bartosz et al. showed that hypoxia inducible factor stabilization leads to increased expression of *VEGF* and *EPO* in the hippocampus and causes lasting improvement of hippocampal memory in healthy mice [31]. Our research also suggests that activation of *HIF-1* appears to have a beneficial role in the hypoperfusion brain as indicated by decreased angiogenesis, and increased oxidative damage and glial activation in *HIF-1α*-knockdown rats. Therefore, *HIF-1α* accumulation induced by 2VO contributes to endogenous neuroprotective responses. Aggravated learning and memory disorders were associated with more severe neuronal damage caused by suppressing HIF-1α.

### 3.3. The Role of HIF-1α in the Regulation of Target Genes

The effect of *HIF-1* is determined by its regulation of target genes during hypoxia or ischemia. Here, we observed that 2VO triggered the increases of protein levels of *HIF-1* target genes (*VEGF*, *GLUT-1*) involved in angiogenesis and energy metabolism, which was in line with the increased accumulation of *HIF-1α* protein. Lentiviral-mediated shRNAi could prevent the increase of protein levels of *HIF-1*-target pro-survival genes by suppressing *HIF-1α* accumulation. However, no significant changes were observed in the expressions of pro-apoptosis genes. Our previous study also found that the tendency of an increase in pro-survival genes was sustained for at least one week, whereas the increase in pro-apoptosis genes was relatively temporary [20]. *HIF-1* may regulate pro-survival genes more than pro-apoptosis genes, explaining why the neuroprotective effects of *HIF-1* prevailed over its pro-death effects during CCH. Thus, it is possible that the reduction in the expression of some pro-survival genes in shRNAi-treated 2VO rats may be attributed to a reduction of neuronal *HIF-1* activity, which results in a suboptimal adaptive response to hypoperfusion that exacerbates the neuronal damage and cognitive impairment.

In addition to *VEGF*, *GLUT-1*, and *EPO*, some other *HIF-1* downstream genes involved in the regulation of energy metabolism, oxidative stress, and mitochondrial integrity may also contribute to the neuroprotective role of *HIF-1*. These genes include manganese superoxide dismutase (*MnSOD*), pyruvate dehydrogenase (*PKD-1*), peroxisome proliferator-activated receptor-c coactivator-1a (*PGC-1*), etc. [5,32]. Further measuring the expressions of these genes may be useful to support the beneficial role of *HIF-1*.

## 4. Materials and Methods

### 4.1. Animals

Rats for this experiment were obtained from Vital River Laboratory Animal Technology Co., Ltd. (Beijing, China). Their use was approved by the Animal Ethics Committee of the Medical School of Wuhan University (WHAE0321178, 21 April 2013). The procedures performed all followed protocols approved by the National Institutes of Health Guide for the Care and Use of Laboratory Animals. 160 male Wistar rates aged 7 weeks and weighing between 220 and 250 g at the outset were obtained. Housing conditions were as follows: group-housing in polypropylene cages, ad libitum access to food and water, a temperature of 22 ± 2 °C, humidity levels of 55% ± 5%, and a twelve-hour artificial dark/light cycle (light from 7:00 a.m.–7:00 p.m.).

### 4.2. Experimental Design

We used RNA interference (RNAi) technology to suppress *HIF-1α* expression in the rat brain. Lentivirus constructs that express green fluorescent protein (GFP) and short-hairpin RNA (shRNA) directed against the *HIF-1α* mRNA (pLVx-GFP-shHIF-1α) were stereotaxic injected into the bilateral hippocampus and bilateral ventricles to knockdown *HIF-1α* gene expression in the hippocampus and some other brain areas. As a negative control, lentivirus constructs that express GFP and shRNA with a scrambled sequence (pLVx-GFP-shCon) were injected in the same way. Rats without stereotaxic injection were used as blank controls.

One week after stereotaxic injection, permanent bilateral common carotid artery occlusion (2-vessel occlusions (2VOs)) was used to induce chronic global cerebral hypoperfusion in the rats. Spatial learning and memory performances were evaluated four weeks after 2VO via the Morris Water Maze (MWM) test. Samples for Western blot assays, quantitative real-time (qRT)-PCR, histopathological and immunohistochemical analyses were obtained from the brains of other rats without performing the MWM test.

**Figure 8:**
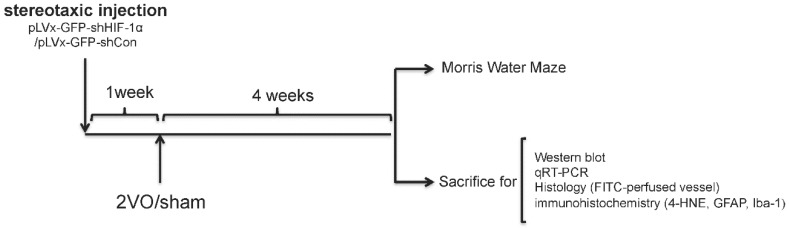
Experimental designs of the present study.

The experimental design is illustrated in Figure 8.

### 4.3. Construction of Lentivirus Vectors

The lentivirus vectors (pLVx-GFP-shHIF-1α and pLVx-GFP-shCon) used in our study were constructed by Land Biology Technology Co., Ltd. (Shenzhen, China).

The *HIF-1α* short hairpin RNA interference (shRNAi) sequence was as follows: 5′-gatccAATCAAAAGCAGTGACGAActcgagTTCGTCACTGCTTTTGATtttttg-3′ (Hif1aBamHI F) and 5′-aattcaaaaaAATCAAAAGCAGTGACGAActcgagTTCGTCACTGCTTTTGATTGg-3′ (Hif1aE croRI R). *HIF-1α* target sequence is 5′-AATCAAAAGCAGTGACGAA-3′, which corresponds to bases 238–256 bp of the *HIF-1α* mRNA (GenBank accession number NM_023459). shRNAi with a random sequence was also designed to use as a control lentivector.

The titers of pLVx-GFP-shHIF-1α vectors is 2.5 × 10^8^ transducing U/mL. Application of pLVx-GFP-shHIF-1α resulted in significantly lower expression of *HIF-1α* mRNA (>70%) in 293T cells. (These results are from Land Biology Technology Co., Ltd.).

Virus suspensions were stored at −80 °C until use and were briefly centrifuged and kept on ice immediately before injection.

### 4.4. Stereotaxic Injection of the Lentiviral Vectors

After anesthesia with 10% chloral hydrate (350 mg/kg, intraperitoneal injection), rats were placed into a stereotaxic frame (RWB Life Science, Shenzhen, China). In order to transfect neurons in as many brain areas as possible, pLVx-GFP-shHIF-1α or pLVx-GFP-shCon was injected into the bilateral hippocampus and bilateral ventricles. Rats without stereotaxic injection were used as blank controls.

Two microliters of pLVx-GFP-shHIF-1α or pLVx-GFP-shCon were injected into the hippocampus using a 10 µL Hamilton syringe with a 33 gauge tip needle (Hamilton). Injection coordinates relative to the bregma were as follows: anteroposterior, −4.00 mm; mediolateral, ±2.5 mm; dorsoventral −3.8 mm below the surface of skullusing coordinates derived from the atlas of Paxinos and Watson (Version 2004). Four microliters of pLVx-GFP-shHIF-1α or pLVx-GFP-shCon were injected into bilateral ventricles. Injection coordinates relative to the bregma were as follows: anteroposterior, −1.00 mm; mediolateral, ±1.5 mm; dorsoventral, −3.6 mm below the surface of dura using coordinates derived from the atlas of Paxinos and Watson (Version 2004).

The injection rate was 0.5 μL/min. At the end of the injection, the needle was maintained in the place for another 5 min and then withdrawn very slowly to prevent backflow of solution.

The accuracy of injection sites was identified by the Evans blue (Sigma; St. Louis, MO, USA) stereotaxic injection directly into the hippocampus and ventricle. The needle track in the ventricle and hippocampus was shown in Figure 1A,B.

Green fluorescent protein (GFP) was observed one week after injection under fluorescence microscope for detection of virus delivery. To test the interference efficiency, the hippocampus was chosen as representative and its *HIF-1α* mRNA level was detected by qRT-PCR one week after injection of lentiviral vectors.

### 4.5. 2VO Surgery

One week after stereotaxic injection, the 2VO surgery to induce CCH was carried out as previously described [33]. Food and water were withheld for one day prior to surgery. Under chloral hydrate (350 mg/kg, i.p.) anesthesia, a midline ventral incision was used to expose the bilateral common carotid arteries. The arteries were carefully separated from the vagal nerves and permanently ligated with silk sutures. The procedure was the same for the sham operations but for the ligation of the common carotid arteries. The surgical wounds were then sutured and the rats were returned to their cages once they had recovered from the anesthesia. Body temperature of the rats was maintained at 37.5 ± 0.5 °C throughout surgery.

### 4.6. Morris Water Maze Performance

Spatial learning and memory were measured using a Morris Water Maze (MWM) four weeks after the 2VO surgery.

The Maze consisted of a 2.0 m-diameter round pool filled to 32 cm deep with 20 ± 1 °C water. The surface area of the tank was divided into four equal quadrants. A platform (9 cm in diameter and 30 cm in height) was placed inside the tank and was not visible. During the entire test, the platform was kept in the same quadrant. The walls of the tank, the water and the platform were made opaque with nontoxic black paint. The surroundings were dimly lit, and the illumination was kept constant throughout the test. A number of orientation cues were placed around the tank to aid the rats in learning the location of the platform. Cued training consisted of four trials per day for the first five days. Each day the rats were released at a different location into the water facing the pool walls. Rats were given 60 s to find the platform without assistance before being guided to it and allowed to rest there for at least 20 s. The escape latencies were recorded during the 5-day cued training after which the platform was removed from the pool and each rat was given one 30-s swim probe trial (day 6); the time spent in the target quadrant in which the platform had previously been located was recorded. Data were acquired using the SMART 2.5 computerized video imaging analysis system (Panlab, Barcelona, Spain).

### 4.7. Western Blot

Immunoblotting analysis kits were obtained from the Keygen Institute of Biotechnology (Keygen, Nanjing, China). Protein samples were extracted from the hippocampus and cortex of the rat-nuclear protein samples for HIF-1α protein analysis, and total proteins for HIF-1 downstream protein analysis. Samples of 40 µg each underwent the following procedure: fractionation with 10% SDS-PAGE; transfer to 0.45-mm PVDF membranes (Invitrogen, Carlsbad, CA, USA) at 200 mA for one hour; staining with Ponceau red to confirm equal loading. For one hour at room temperature the blots were blocked with 5% nonfat milk in Tris-buffered saline containing 0.1% Tween 20 (TBST). Overnight incubation (at 4 °C) of the membranes with primary antibodies (anti-HIF-1α, anti-VEGF, anti-GLUT-1, and anti-EPO, 1:1000 dilution in TBST, BD Transduction Laboratories, San Diego, CA, USA; anti-β-actin, 1:2000 in TBST, Santa Cruz, Dallas, TX, USA) was performed. The membranes were then washed three times with TBST and a 30 min incubation with peroxidase-conjugated secondary antibodies (anti-mouse: 1:3000 in TBST, KPL, Gaithersburg, MD, USA) was performed. The membranes were again washed three times in TBST and chemiluminescence (SuperSignal West Pico, Pierce, Rockford, IL, USA) was used to visualize labeled proteins. Optical density analysis (HPIAS 2000, Tongji Qianping Company, Wuhan, China) was used for semi-quantification of immunoreactive bands on X-ray film with all densities normalized to β-actin.

### 4.8. qRT-PCR

qRT-PCR was used to observe the mRNA levels of HIF-1α and its downstream genes after 2VO and HIF-1α knockdown.

The Trizol method (Invitrogen, Carlsbad, CA, USA) was used on total RNA extracted from the hippocampus and cortex. A First Strand cDNA Synthesis kit (TOYOBO, Kita-ku, Japan) was used on 4 µg of RNA to reverse transcribe it to cDNA, of which 0.2 µg was then amplified using a qRT-PCR kit. The reaction used THUNDERBIRD SYBR qPCR Mix (TOYOBO, Kita-ku, Japan). The gene-specific primers used can be seen in Table 1. The following PCR cycles were applied: initial denaturation at 50 °C for 2 min, then 95 °C for 2 min; 40 cycles of 15 s at 95 °C, annealing at 58 °C for 15 s, and extension at 72 °C for 45 s; and extension at 72 °C for 10 min. β-actin was used as an endogenous control and reactions were all performed in triplicate. The formula (2^-ΔΔ*C*t^) was applied to express changes in mRNAs.

### 4.9. Density of Capillaries

To identify the network of capillaries and evaluate angiogenesis after 2VO and HIF-1α knockdown, rats were anesthetized with 10% chloral hydrate (350 mg/kg, i.p.) and transcardially perfused at 4 weeks after 2VO with 5 mL FITC-dextran (2 × 10^6^ molecular weight, Sigma; St. Louis, MO, USA; 50 mg/mL) at a perfusion pressure of 120–140 mmHg. The perfused vessels can be marked by FITC-dextran. The brains were removed, immersed in 4% paraformaldehyde at 4 °C for 48 h. Coronal sections (150 µm thick) were made by vibratome microtome, and fluorescence images were obtained using a laser scanning confocal microscope (ZEISS LSM 700, Carl Zeiss AG, Germany) under 488 nm wave excitation. Cortex, hippocampus and corpus callosum areas of brain were scanned under a ×40 objective lens. The full-focused images were generated from the Z-stack images using ZEISS application (Carl Zeiss AG, Germany). A FITC-perfused vessel (<12 µm) that is separated from adjacent vessels was regarded as a single capillary and counted in number. Capillary density was expressed as the number of capillaries per square millimeter.

### 4.10. Immunohistochemical Staining

Immunohistochemical staining was used to detect oxidative damage and inflammatory-related activation of glial cells in brain.

A 10% chloral hydrate (350 mg/kg, i.p.) anaesthetic was administered to the rats. Once they were perfused transcardially using 4% buffered PFA (pH 7.4), the brain was removed. It was stored for at least 24 h in fixative then embedded in paraffin wax and sectioned coronally (4 µm thickness). Once blocked, the brain slices were incubated overnight at 4 °C with the primary antibodies used for immunohistochemistry. In this procedure, the primary antibodies used were directed against the lipid peroxidation marker 4-hydroxynonenal (*4-HNE*; Millipore Chemicon International, Temecula, CA, USA, 1:500 dilution), the astrocyte marker glial fibrillary acidic protein (*GFAP*; GeneTex, Irvine, CA, USA, 1:250 dilution) and the microglial marker ionized calcium-binding adapter molecule 1 (*Iba-1*; Abcam, Cambridge, MA, USA, 1:100 dilution). Secondary antibodies were then used to wash and incubate the brain sections for one hour at room temperature. A solution of 50 µL diaminobenzidine solution was used to develop the reaction products and was also used to stain similar brain sections that were not treated with primary antibodies as a negative control. Image-Pro Plus 6.0 image analysis software (Media Cybernetics, Silver Spring, MD, USA) was used to magnify to 100× the sections immunostained for *GFAP* or *Iba-1*. The number of immunopositive cells were then counted in three visual fields (cells/mm^2^) in the corpus callosum, the cerebral cortex, and certain subregions (CA1, CA2, CA3, and DG) of the hippocampus. For cells positively immunostained for *4-HNE*, mean integral optical density (IOD) values were determined. To achieve this, five slices with the greatest differences in staining intensity were identified. They were assessed according to the manual to choose the most appropriate parameters to ensure the largest ratio of hippocampal pyramidal cells to other background cells. These parameters were applied to analyze all stained slices. Differences between groups were determined using GraphPad Prism 5.0 software [1].

### 4.11. Statistical Analyses

All results are presented as mean ± SEM. The statistical analyses were performed via GraphPad Prism 5.0 software (GraphPad Software, Inc., La Jolla, CA, USA). Differences in escape latencies were analyzed with a two-way repeated measures ANOVA, and Bonferroni post-tests or unpaired student’s *t* tests were used for post hoc multiple treatment comparisons. All other measurements were analyzed using one-way ANOVAs followed by Bonferroni post hoc tests. Statistical significance was defined as *p* < 0.05.

## 5. Conclusions

In conclusion, the results of our study provide evidence that *HIF*-mediated pro-survival responses are dominant in rats with CCH. The activation of *HIF-1* is part of a homeostatic response aimed at coping with the deleterious effects of CCH.

## Figures and Tables

**Table 1:** Primers.

Gene	Orientation	Sequence (5’–3’)	Amplicon Size (bp)
*HIF-1α*	Forward	AAGCACTAGACAAGCTCACCTG	75
	Reverse	TTGACCATACGCTGTCCAC	
*VEGF*	Forward	TGTGAGCCTTGTTCAGAGCG	252
	Reverse	GACGGTGACGATGGTGGTGT	
*EPO*	Forward	CACCCTGCTGCTTTTACTATCCTT	139
	Reverse	CATTGTGACATTTTCTGCCTCCT	
*GLUT-1*	Forward	CCGCTTCCTGCTCATCAATC	122
	Reverse	TCATCTGCCGACCCTCTTCT	
*BNIP-3*	Forward	ATGGGATTGGTCAAGTCGGC	205
	Reverse	CTTCCAATGTAGATCCCCAAT	
*Noxa*	Forward	ACGTGGAGTGCACCGGACAT	168
	Reverse	TTTCTGCCGTAAATTCACTT	
*NIX*	Forward	TTCAGACACCCTAAGCGTGC	176
	Reverse	GCAGAAGGTGTGCTCAGTCGT	
β*-actin*	Forward	CGTTGACATCCGTAAAGACCTC	110
	Reverse	TAGGAGCCAGGGCAGTAATCT	
